# Recombinant Human Decorin Normalizes the Active Features of Breast Cancer-Associated Fibroblasts

**DOI:** 10.3390/cells15030311

**Published:** 2026-02-06

**Authors:** Wafaa A. Aljagthmi, Ayodele A. Alaiya, Maha Daghestani, Falah H. Al-Mohanna, Abdelilah Aboussekhra

**Affiliations:** 1 Research Laboratories, King Faisal Specialist Hospital and Research Center, Riyadh 11211, Saudi Arabia; 2 Department of Zoology, College of Science, King Saud University, Riyadh 11451, Saudi Arabia; 3 Department of Comparative Medicine, King Faisal Specialist Hospital and Research Center, Riyadh 11211, Saudi Arabia

**Keywords:** breast cancer, CAFs, DCN, cancer stem cells

## Abstract

**Highlights:**

**What are the main findings?**

- rhDCN can normalize the active features of breast cancer-associated fibroblasts.
- rhDCN-dependent normalization of CAFs is persistent.

**What are the implications of the main findings?**

- Utilization of rhDCN to target active CAFs in breast tumors.
- rhDCN could be used for the treatment of various types of cancer through the inhibition of the pro-carcinogenic effects of CAFs.

**Abstract:**

Cancer-associated fibroblasts (CAFs), the major constituent of the tumor microenvironment, are considered the most active cells and key contributors to tumor resistance, recurrence, and metastasis. Therefore, we have investigated here the potential normalization of the active features of breast CAFs with decorin (DCN), a small leucine-rich proteoglycan that acts as an oncogene suppressor. We have first shown that rhDCN modulates the expression of a plethora of proteins involved in different signaling pathways, including STAT3/NF-κB and ERK. Consequently, rhDCN repressed the important active CAF biomarkers α-SMA, IL-6, and SDF-1 through inhibition of the STAT3/AUF-1 pathway, in cells grown as 2D and 3D cultures. Furthermore, rhDCN had a strong downregulation effect on FAP-α, a key biomarker of active CAFs, and suppressed their proliferative and invasive capacities through upregulation of p16 and p21, and downregulation of MMP-2 and MMP-9. Furthermore, rhDCN suppressed the paracrine effects of active CAFs in promoting epithelial-to-mesenchymal transition (EMT) and cancer stem cells in breast cancer cells, both in vitro and in orthotopic tumor xenografts. Importantly, rhDCN-related normalization of active CAFs features was persistent through cellular passaging, and was not accompanied by cytotoxicity. Together, these findings have revealed rhDCN as a promising anti-breast cancer therapeutic cytokine through suppression of the non-cell-autonomous cancer-promoting effects of active CAFs.

## 1. Introduction

In solid tumors, including breast carcinomas, cancer cells are part of a complex ecosystem where the tumor microenvironment (TME) plays important roles in tumor onset, spread, and response to various types of treatments [1,2]. Within the TME, the functional interplay between cancer cells and active stromal cells, such as fibroblasts, is orchestrated by a plethora of signaling molecules, including cytokines. Cancer-associated fibroblasts represent the most active cellular components in TME, and they are closely associated with tumor initiation and development [3,4]. Furthermore, active CAFs fuel tumors, promote their growth, and promote the formation of cancer stem cells through the secretion of various types of cytokines [5,6]. Cytokines are small secreted signaling proteins produced by both normal cells and cancer cells, and are involved in the various steps of the immune responses, development, growth, and tissue repair. Cytokines are important elements in health and disease; therefore, it is of utmost importance to explore their power for therapeutic use [7,8]. Increasing evidence shows that cytokines are heavily involved in multiple aspects of cancer development and may be involved in regulating both pro- and anti-tumorigenicity [7,9]. In cancers, active CAFs secrete high levels of pro-carcinogenic cytokines such as IL-6, IL-8, and SDF-1, while the anti-carcinogenic cytokines such as decorin (DCN) are repressed [5,6,10].

Decorin (DCN), a small leucine-rich proteoglycan oncosuppressor protein, is present in the extracellular matrix of different tissues [11,12]. In addition to the multiple biological functions of DCN, which include cell proliferation, apoptosis, autophagy, and angiogenesis, several studies have revealed the important role of DCN in inhibiting tumor growth and metastasis [13,14,15]. Indeed, decorin is an efficient inhibitor of several tyrosine kinase receptors, including IGFR, HER2, VEGFR2, and TLR [16]. Deficient expression of DCN in animal models contributed to tumorigenesis and the epithelial-to-mesenchymal transition (EMT) process [17]. Furthermore, DCN can bind and sequester TGF-α, which reduces fibrosis and inflammation [18]. These findings indicated that DCN has anti-cancer therapeutic potential [19]. Recently, we have shown that DCN downregulation activates normal breast stromal fibroblasts (NBFs), while ectopic expression of DCN suppresses CAFs active features, including their paracrine pro-tumorigenic effects: EMT, stemness, and angiogenesis [10].

We have explored here the anti-carcinogenic effects of the recombinant human DCN (rhDCN) protein on the active breast CAFs. We have demonstrated that rhDCN upregulates endogenous DCN and normalizes the active features of CAFs both in vitro and in orthotopic tumor xenografts.

## 2. Materials and Methods

### 2.1. Cells, Cell Culture, and Reagents

Cancer-associated fibroblasts were isolated from surgical tissue samples collected at KFSHRC, and all patients were consented as previously described [20]. CAF cells were cultured in media composed of: M199 medium, Ham’s F12, 10% FBS, and 1% 100x-Antibiotic-Antimycotic (Gibco, Grand Island, NY, USA). MDA-MB-231 and MCF-7 breast cancer cells were purchased from ATCC, and were cultured in media composed of RPMI 1640 medium, 5% FBS, and 1% Antibiotic-Antimycotic 100X (Gibco, Grand Island, NY, USA). Cells were cultured at 37 °C in a humidified incubator with 5% CO_2_. Recombinant human decorin (rhDCN) was obtained from R&D Systems in a lyophilized form and was reconstituted in sterile PBS according to the manufacturer’s instructions (Cat#143-DE) (Minneapolis, MN, USA).

### 2.2. Direct Co-Culture

CAF and MCF-7 cells were co-cultured in a transwell tissue culture plate (with an insert membrane) for 24 h. While CAFs were seeded in the upper chamber (upper surface of the insert), MCF-7 cells were seeded in the lower chamber (underside of the insert) of the transwell plate, which allows cells to grow toward each other and form contact through the porous membrane. Only MCF-7 cells were collected. These experiments were repeated 3 times. 

### 2.3. RNA Purification and qRT-PCR

The RNeasy Mini Kit was used to prepare total RNA, following the manufacturer’s instructions (Qiagen, Manchester, UK), and then the concentrations were determined with a Spectrophotometer (NanoDrop ND-1000, Thermo Scientific, Waltham, MA, USA). Random primers were utilized to prepare cDNA and amplified by RT-PCR. The amplification reactions were carried out by the Light Cycler 96 Real-Time PCR System (Roche, Mannheim, Germany). As an internal control GAPDH was utilized, and relative gene expressions were calculated from the threshold cycle (Ct) values using Light Cycler^®^ 96 SW 1.1 software. Primer sequences are shown in the Supplementary Table S1. These experiments were carried out in triplicate and repeated 3 times.

### 2.4. Serum-Free Conditioned Media Preparation

Cells were first incubated for 24 h in serum-free medium (SFM), and then the resulting supernatants were harvested, filtered, centrifuged, aliquoted, and then stored at −80 °C.

### 2.5. Cell Lysate Preparation and Immunoblotting

Cells were centrifuged, and the pellets were resuspended in the lysis buffer RIBA (Sigma-Aldrich, St. Louis, MI, USA) containing protease inhibitors (Roche). The proteins obtained after centrifugation of the cell lysates were stored at −80 °C. SDS-PAGE was followed by protein transfer to PVDF membranes (Bio-Rad, Hercules, CA, USA), which were blocked in 5% powdered skimmed milk in TBST for 1 h. Primary antibodies were next applied and incubated overnight. Then, PVDF membranes were incubated in secondary antibodies for 1 h. Following the manufacturer’s instructions (Thermo Fisher Scientific, Waltham, MA, USA), a chemiluminescence detection method was used to visualize the secondary antibody. Antibodies directed against pNF-κB p65 (Ser536), NF-κB p65 (D14E12), STAT3 (124H6), p-STAT3 (Tyr705), MMP-9, MMP-2, p-ERK (T202/Y204), and ERK [137F5] were purchased from Cell Signaling Technology (Danvers, MA, USA). α-SMA [polyclonal], TGF-β1 [2Ar2], SDF-1 [polyclonal], Vimentin [RV202], N-cadherin [13A9], E-cadherin [24E10], IL-6 [Polyclonal], FAP-α [polyclonal], and Twist1 [10E4E6] were purchased from Abcam (Cambridge, UK). GAPDH [FL-335], CD24 (SN3), p16 [F-12], and p21 [C-19] were purchased from Santa Cruz Biotechnology (Santa Cruz, CA, USA). CD44 [polyclonal] and ALDH1 [1G6] were purchased from Sigma-Aldrich (Burlington, MA, USA). AUF1 [polyclonal] was purchased from Millipore. DCN (115402) from R&D Systems (Minneapolis, MN, USA). These experiments were repeated several times.

### 2.6. Cell Proliferation and Invasion

The RTCA-DPxCELLigence System (ACEA Biosciences, Santa Clara, CA, USA) was utilized following the instructions of the manufacturer. For invasion assays, 20 μL of Matrigel (BD Biosciences, Franklin Lakes, NJ, USA) was used to precoat microporous membranes, which were then incubated at 37 °C for 4 h. Then, 1 × 10^5^ cells in 100 μL of SFM were seeded into the upper chambers of a CIM-plate, while 200 μL of complete medium was added to the lower chambers. For proliferation, cells were seeded into an E-plate containing adequate medium. The RTCA-DP software (version 1.2.1) was used to analyze the obtained results. These experiments were carried out in triplicate and were repeated several times.

### 2.7. Three-Dimensional Spheroid Assay

Spheroids were generated by seeding 2.000 cells per well in ultralow-attachment 96-well plates, in stem cell-specific medium for several days. Only spheroids with a diameter larger than 100 μm were counted by an inverted microscope (FLoid Cell Imaging Station, OPTICA, Washington, DC, USA). These experiments were performed in triplicate and repeated several times.

### 2.8. Three-Dimensional (3D) Cell Culture

3D insert scaffolds were utilized to culture cells as 3D in a 6-well plate for 2–3 days. Cells were then either sham-treated or treated with rhDCN (0.1 μg/mL) for 24 h. Cells were collected, and RNA was extracted and amplified by qRT-PCR. These experiments were carried out in triplicate and repeated several times.

### 2.9. Immunofluorescence Assay

Cells were fixed with paraformaldehyde (4% in PBS) for 20 min at room temperature, followed by non-permeabilization blocking buffer for 60 min. Primary antibody was used overnight at 4 °C, followed by washing and incubation with fluorophore-conjugated secondary antibody (ex, Alexa 594) for 1 h; cell nuclei staining was performed with 4,6-diamidino-2-phenylindole. Images were captured using the Fluorid Cell Imaging Station (Life Technologies, Waltham, CA, USA).

### 2.10. Cytotoxicity Assay

The WST-1 reagent (Roche) was used to test cell viability according to the manufacturer’s instructions. A microplate reader (Bio-Rad) was utilized to measure the absorbance at 450 nm. These experiments were carried out in triplicate and repeated several times.

### 2.11. Orthotopic Tumor Xenografts

All animal experiments were approved by the KFSH&RC Institutional Animal Care and Use Committee (ACUC) under RAC#2180018 (1 August 2022) and conducted in accordance with relevant national and international guidelines. Orthotopic breast tumor xenografts were generated by co-injecting MDA-MB-231 cells (2 × 10^6^) with either CAF-118-rhDCN or CAF-118-CTRL cells (4 × 10^6^) under the nipple of each female nude mouse (Mus musculus, Nu/J, 6 weeks, 25 g, from the Jackson Laboratories) (n = 10, 5 in each subgroup). The animals were numbered from 1 to 10 and were kept in cages in an animal facility; animals that developed necrosis were excluded. Tumor volumes were measured twice weekly using a caliper. At the end of the experiment, tumors were excised and snap-frozen in liquid nitrogen for subsequent analyses. During the experiment, the scientist and the technician were aware of the group allocation

### 2.12. Proteomics Analysis LC/MS

Proteins (100 µg) extracted in the presence of protease inhibitors were digested, and the resulting peptides were analyzed by mass spectrometry (Waters Scientific, Manchester, UK). The obtained results were acquired in a range of m/z 50–2000 Da for a 120 min gradient acquisition run time using data-independent acquisition (MS^E^)/ion mobility separation experiments (HDMS^E^). All samples were analyzed in triplicate runs using the Mass Lynx platform (Version 4·1, SCN833). Progenesis LC/MS (QI fP, V 3.0) (Nonlinear, Newcastle, UK) was used for all automated data processing and database searching against UniProt/Swiss Prot human-specific (*Homo sapiens*) protein sequences.

### 2.13. Functional Pathways and Network Analyses

Functional, canonical pathway, and protein–protein interaction network analyses were performed using Ingenuity Pathways Analysis (IPA) (QIAGEN, Redwood City, CA, USA). Differentially expressed proteins (DEPs) were mapped to their corresponding objects in the Ingenuity pathway knowledge base and gene interaction networks. A right-tailed Fisher’s exact test was used to calculate *p*-values. All statistical tests were two-sided, and a *p*-value < 0.05 was considered statistically significant.

### 2.14. Statistical Analysis and Quantification

Statistical analysis was carried out using a two-tailed unpaired Student’s *t*-test performed in the GraphPad Prism software (4.7.8). *p*-values ≤ 0.05 were considered statistically significant. The immunoblotting resulting bands were quantified using ImageJ (version 1.53). Proteomics-related statistical analysis was detailed in the relevant sections.

## 3. Results

### 3.1. rhDCN Suppresses Several Cancer-Related Pathways in CAF Cells

**Figure 1:**
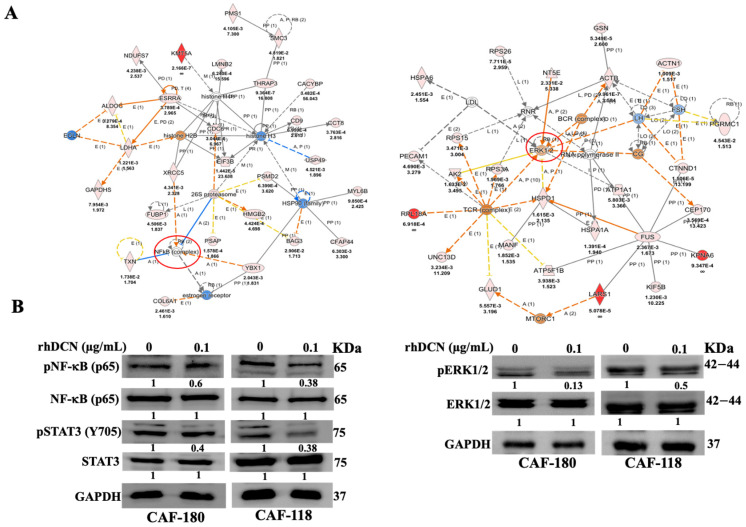
rhDCN inhibits several CAF-related activation pathways. (**A**), Ingenuity Pathway Analysis (IPA) of the 146 overlapping proteins identified in the pairwise comparisons of Rx-DCN-treated cells from two CAF cells (CAF-118 and CAF-180). The key upstream regulators at the core of these networks are: (**Left panel**): NF-κB, 26S Proteasome, and Histone proteins; (**Right panel**): ERK1/2, RNA Polymerase, and TCR Complex. The figures were partially generated using the licensed Ingenuity Pathway Analysis software (Qiagen; www.qiagen.com). (**B**), Cells were either sham-treated (control) or exposed to rhDCN as shown, and then whole-cell lysates were prepared and used for immunoblotting analysis utilizing antibodies targeting the indicated proteins. Band intensities were normalized to GAPDH and presented as fold changes relative to the respective controls. Phosphorylated protein levels were quantified and normalized to their corresponding non-phosphorylated forms.

DCN is a secreted cytokine with anti-cancer effects [12]. Since CAFs are active pro-carcinogenic cells, we decided to investigate the possible normalization of active breast CAFs using recombinant human decorin (rhDCN). Therefore, we have first investigated the effect of rhDCN on protein expression in CAF cells. CAF-118 and CAF-180 cells were either sham-treated or exposed to rhDCN (100 ng/mL) for 24 h, and then whole-cell lysates were prepared and subjected to one-dimensional label-free quantitative global proteomic analysis. Unsupervised hierarchical clustering of differentially expressed proteins (DEPs) revealed 523 and 253 proteins that were significantly differentially expressed (*p* < 0.05, fold change >1.5) in CAF-118 and CAF-180 cells, respectively (Supplementary Figure S1A). The Venn Diagram depicted in Supplementary Figure S1B shows 146 proteins commonly differentially expressed across both cell cultures. The unsupervised hierarchical clustering of the 146 DEPs is depicted in Supplementary Figure S1C. Figure 1A shows that among the 146 proteins, treatment with rhDCN affected several important cancer-related proteins and pathways, which are also deregulated in both CAF cell cultures. These include NF-κB and ERK1/2 signaling (Figure 1A). Indeed, Figure 1B shows a clear rhDCN-dependent decrease in the level of the active/phosphorylated forms of NF-κB and ERK1/2, while their basal levels were not affected. This shows that rhDCN can inhibit the phosphorylation/activation of both signaling molecules NF-κB and ERK1/2. Since the NF-κB pathway is interrelated with the STAT3 pathway, we also examined the effect of rhDCN on STAT3 and showed inhibition of this transcription factor (Figure 1B).

### 3.2. rhDCN Suppresses the Expression of Active CAF Biomarkers

**Figure 2:**
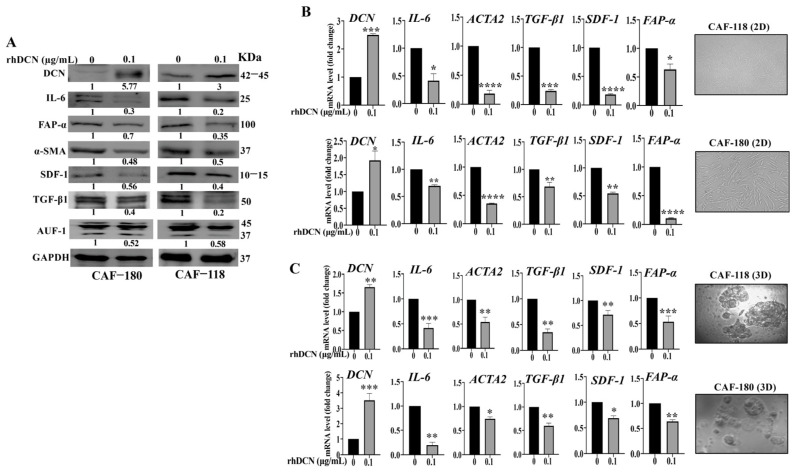
rhDCN suppresses the expression of active CAF biomarkers. CAF-180 and CAF-118 cells were cultured as a monolayer (2D) in complete medium containing either PBS (0) or rhDCN (0.1 µg/mL) for 24 h. (**A**), Whole-cell lysates were collected and utilized for immunoblotting assays. Band intensities, normalized to GAPDH, represent fold changes relative to controls. The levels of the phosphorylated form of proteins were quantified and normalized relative to their corresponding non-phosphorylated forms. (**B**), Total RNA was extracted from cells treated as shown, and then the mRNA levels of the indicated genes were quantified by qRT-PCR. Error bars represent mean ± SD (n = 3). * *p* ≤ 0.05, ** *p* ≤ 0.01, *** *p* ≤ 0.001, **** *p* ≤ 0.0001. Pictures show cells grown as a monolayer (2D). (**C**), CAF-180, and CAF-118 cells were cultured as 3D, and then were treated either with rhDCN (0.1 μg/mL) or were sham-treated (0) for 24 h. Purified RNA was amplified by qRT-PCR. Error bars represent mean ± SD (n = 3). * *p* ≤ 0.05, ** *p* ≤ 0.01, *** *p* ≤ 0.001. Pictures show cells grown in 3D cell culture.

Next, we investigated the rhDCN effect on the major features of active CAFs. CAF-180 and CAF-118 cells were either sham-treated or exposed to rhDCN (0.1 µg/mL) for 24 h. Prepared protein extracts were used for immunoblotting analysis, with GAPDH serving as an internal control. The level of endogenous DCN was increased in CAF-118 and CAF-180 cells (3-fold and 5.7-fold, respectively) in response to rhDCN compared to the controls (Figure 2A). Concomitantly, a decrease in the level of the FAP-α, α-SMA, SDF-1, TGF-β, and IL-6 proteins was also observed in cells treated with rhDCN compared to controls (Figure 2A). Similarly, the mRNA levels of the coding genes *DCN*, *ACTA2*, *TGF-β*, *CXCL-12*, and *IL-6* were also reduced (Figure 2B). This indicates that rhDCN can suppress the biomarkers of active CAFs. This rhDCN-dependent downregulation was confirmed on the endogenous DCN and the other coding genes (ACTA2, TGF-β, CXCL-12, and IL-6) when CAF-180 and CAF-118 cells were grown in 3D cultures (Figure 2C).

To further confirm the inhibition effect of rhDCN on the major CAF biomarker FAP-α, we tested the effect of rhDCN on the expression of FAP-α in situ by immunofluorescence. Supplementary Figure S2 shows that the level of FAP-α was markedly reduced in CAF cells that were treated with rhDCN compared to the controls. The histogram shows that the intensity of FAP-α expression decreased in CAF180-rhDCN (3-fold) and in CAF118-rhDCN (4-fold) (Supplementary Figure S2). These results show that rhDCN suppresses active CAFs biomarkers. This prompted us to ask how the cytokine DCN controls all these active CAF biomarkers. To this end, we tested the effect of rhDCN on the expression level of AUF-1, the main regulator of all these markers [21]. Figure 2A shows that AUF-1 was downregulated in CAF-180 cells (1.9-fold) and in CAF-118 cells (1.7-fold) that were treated with rhDCN relative to controls. This effect was mediated via the downregulation of STAT3, which is the AUF-1 upstream activator [21] (Figure 1B). This indicates that rhDCN inhibits the phosphorylation/activation of STAT3, leading to AUF-1 downregulation, which elucidates the role of DCN in suppressing active CAF biomarkers.

### 3.3. rhDCN Inhibits the Invasive and Proliferative Abilities of CAFs

**Figure 3:**
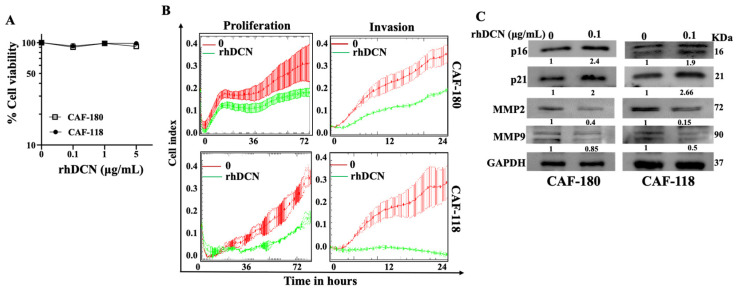
rhDCN is not cytotoxic but inhibits the proliferative and invasive capacities of CAF cells. (**A**), CAF-180 and CAF-118 cells were seeded in 96-well plates (5.10^3^) and were either sham-treated (PBS, 0) or challenged with recombinant human DCN (rhDCN) at different concentrations (0.1, 1, and 5 µg/mL), and then the cells were incubated for 72 h. Cytotoxicity was tested using the WST-1 assay. Error bars represent mean ± SD; n = 3. (**B**), Cellular proliferation/invasion capacities were assessed using the RTCA-DPxCELLigence System. Data are representative of different experiments performed in triplicate. (**C**), Immunoblotting analysis. The numbers are fold changes relative to the control (0) after correction against GAPDH.

Next, we have examined the cytotoxic effect of DCN on CAF cells. To this end, CAF-180 and CAF-118 cells were seeded in a 96-well plate (5.10^3^), and then the cells were treated with rhDCN at different concentrations (0, 0.1, 1, and 5 µg/mL) for 72 h. The cytotoxicity was determined using the WST1 assay. Figure 3A shows that exposing CAF-180 and CAF-118 cells to increasing concentrations of rhDCN did not affect the cell viability of these cells. This means that DCN has no cytotoxic effect on the CAF cells even at high doses.

We have next examined the effect of rhDCN on CAF proliferation and invasion abilities. CAF-180 and CAF-118 cells were challenged with rhDCN (0 and 0.1 µg/mL), and then cell invasion and proliferation capacities were assessed using the RTCA-DP xCELLigence System. Figure 3B shows a reduction in the invasive and proliferative abilities of CAF-180 and CAF-118 cells when they were treated with rhDCN (0.1 µg/mL) compared to the controls (CTRL). This indicates that rhDCN inhibits the invasive and proliferative abilities of breast CAFs.

To confirm the effect of DCN on the proliferative and invasive capacities of active CAFs at the molecular level, we examined the effect of rhDCN on important proliferative and invasive factors. Figure 3C shows that the levels of p16 and p21, two tumor suppressor and cell cycle checkpoint proteins, increased in rhDCN-treated CAF cells relative to controls. However, the expression of MMP-2 and MMP-9, which are pro-invasive and tumor growth proteins [22], decreased in rhDCN-treated CAFs as compared to controls (Figure 3C). These results show that rhDCN inhibits the proliferative and invasive capacities of CAF cells.

### 3.4. rhDCN Suppresses the Paracrine Pro-Carcinogenic Effects of Active CAFs

**Figure 4:**
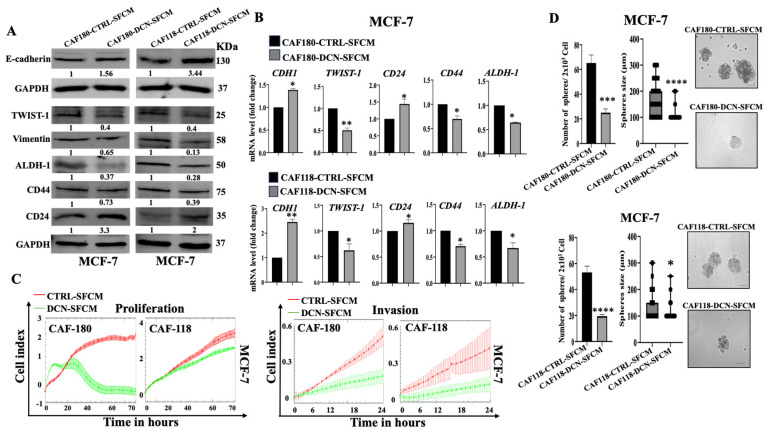
rhDCN suppresses the pro-carcinogenic effects of breast CAFs. Serum-free medium (SFM) was added to CAF180-CTRL, CAF118-CTRL, or CAF180-rhDCN, CAF118-rhDCN cells and incubated for 24 h, and then SFCM were collected (CAF180-CTRL-SFCM and CAF180-rhDCN-SFCM) and (CAF118-CTRL-SFCM and CAF118-rhDCN-SFCM) and were used to treat MCF-7 cells for 24 h. (**A**), Immunoblots. The numbers under bands represent fold changes relative to respective controls (CAF180-CTRL-SFCM or CAF118-CTRL-SFCM) post-correction against GAPDH. (**B**), qRT-PCR, Error bars represent mean ± SD (n = 3). * *p* ≤ 0.05, ** *p* ≤ 0.01. (**C**), The invasive and proliferative capacity of cells was assessed using the RTCA-DP xCELLigence System. (**D**), Cells (2 × 10^3^) were cultured in stem cell-specific medium in an ultralow-attachment 96-well plate. The tumorspheres (≥100 μm) were counted. (**Right panels**), Photographs of the formed tumorspheres. Scale bar = 100 μm. (**Left panels**), Histograms showing the number of counted tumorspheres. Error bars represent mean ± SD; n = 3. * *p* ≤ 0.05, *** *p* ≤ 0.001, **** *p* ≤ 0.0001.

To confirm rhDCN-dependent normalization of active CAFs, we sought to test the effect of rhDCN on the cancer-promoting potential of active CAFs. To this end, CAF-180 and CAF-118 cells were either exposed to rhDCN (0.1 µg/mL) or sham-treated for 24 h, and then the cells were re-incubated in SFM for an extra 24 h. The resulting SFCM: CAF180-CTRL-SFCM, CAF180-rhDCN-SFCM, CAF118-CTRL-SFCM, and CAF118-rhDCN-SFCM were collected and used to treat MCF-7 breast cancer cells for 24 h. The immunoblotting analysis shows that the SFCM from rhDCN-pretreated CAFs inhibited the EMT process in MCF-7 cells through an increase in the expression of the epithelial marker (E-cadherin) and a decrease in the level of the mesenchymal markers (Twist and Vimentin) relative to controls (Figure 4A). This EMT inhibitory effect was confirmed at the mRNA level of the E-cadherin (*CDH1*) and *TWIST* coding genes (Figure 4B). To confirm the anti-EMT effect, we tested the effect of SFCM obtained from CAF-CTRL and CAF-rhDCN on the proliferative and invasive capacities of MCF-7 cells, which were first treated with CAF-SFCMs, and then were seeded in a CIM plate and an E-plate and were incubated for 24 h (invasion) or 72 h (proliferation). Figure 4C shows that CAF-rhDCN-SFCMs inhibited the proliferative and invasive abilities of MCF-7 breast cancer cells compared to controls (CAF-CTRL).

In addition, we examined the effect of CAF-rhDCN-SFCMs on the stemness features of MCF-7 cells. Figure 4A shows that the levels of the stemness markers ALDH-1 and CD44 were decreased, while the level of CD24 was increased in MCF-7 cells that were pre-treated with CAF180-rhDCN-SFCM and CAF118-rhDCN-SFCM as compared to their respective controls. These results were confirmed at the mRNA levels (Figure 4B). To confirm rhDCN-dependent targeting of CSCs, SFCM-treated MCF-7 cells were cultured in an ultralow-attachment 96-well plate in stem cell-specific medium. After 10 days of incubation, tumorspheres with a size ≥ 100 μm were counted. Figure 4D shows a decrease in the spheroid number in MCF-7 cells treated with CAF-rhDCN-SFCMs compared to controls. In addition, the treated cells demonstrated a decrease in the spheroid size compared to controls (Figure 4D). This indicates that rhDCN inhibits the paracrine CAF-dependent promotion of stemness in breast cancer cells. To confirm this, the rhDCN-treated CAF cells (CAF-180/118) were directly co-cultured with MCF-7 breast cancer cells using a trans-well tissue culture plate for 24 h. Total RNA was prepared from MCF-7 cells, and then the mRNAs of EMT and stemness genes were amplified using qRT-PCR. Supplementary Figure S3 shows that rhDCN-CAF cells suppressed the EMT process in MCF-7 cells through upregulation of the epithelial marker E-cadherin-coding gene (*CDH1*) and downregulation of the mesenchymal marker N-cadherin-coding gene (*CDH2*). In addition, the stemness features of MCF-7 cancer cells were also inhibited when directly co-cultured with CAF-rhDCN cells (Supplementary Figure S3). The levels of the stemness-coding genes *ALDH-1* and *CD44* decreased, while the *CD24* level increased, which indicates the inhibition of the pro-stemness capacity of CAF cells that were treated with rhDCN (Supplementary Figure S3).

### 3.5. The Inhibitory Effect of rhDCN on CAF Cells Is Persistent

**Figure 5:**
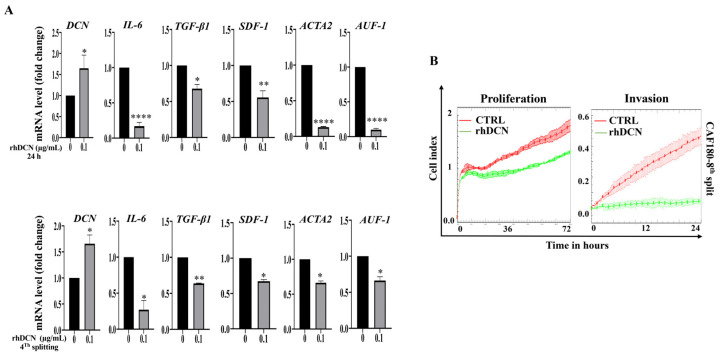
The inhibitory effect of rhDCN on CAF cells is persistent. CAF-180 cells were cultured in complete medium containing or not rhDCN (0.1 µg/mL) for 24 h. Each plate was split 1 in 2 (one half was used to extract RNA, and the other half was re-cultured with complete medium and was split similarly three times). (**A**), Extracted RNA was amplified and quantified by qRT-PCR. Error bars represent mean ± SD (n = 3). * *p* ≤ 0.05, ** *p* ≤ 0.01, **** *p* ≤ 0.0001. (**B**), Cellular proliferative and invasive abilities were assessed using the real-time cell analysis (RTCA-DPxCELLigence System). Data represent independent experiments performed in triplicate.

Since the activation of stromal fibroblasts is a persistent process even in the absence of the activating mechanism, we decided to test the persistence of rhDCN-related normalization of active CAFs. Therefore, cells were cultured in complete medium either alone or containing rhDCN (0.1 µg/mL) for 24 h (CAF180-CTRL, CAF180-rhDCN, CAF118-CTRL, and CAF118-rhDCN). Then, each plate was split into 2 (one half was used to extract RNA, and the other half was re-cultured with complete medium, and then was split three times). Total RNA (24 h and 4th splitting) was prepared, and then specific mRNAs were amplified using qRT-PCR. Figure 5A and Supplementary Figure S4A show that the DCN upregulation following 24 h of treatment was maintained even after 4-times splitting of cells in both cell cultures. Interestingly, rhDCN-dependent inhibitory effect on the CAF biomarkers *IL-6*, *TGF-β*, *CXCL-12*, *ACTA2*, and *AUF-1* was also maintained at the 4th split of cells (Figure 5A and Supplementary Figure S4A). This indicates that rhDCN-dependent DCN upregulation and inhibition of the active CAF biomarkers are persistent. This was confirmed by showing that rhDCN-dependent inhibition of the proliferative and invasive capacities of active CAFs was maintained after 8-times splitting of cells (Figure 5B and Supplementary Figure S4B). This shows that rhDCN-dependent normalization of active CAFs is persistent.

### 3.6. rhDCN-Treated CAFs Inhibit Tumor Growth in Orthotopic Tumor Xenografts

**Figure 6:**
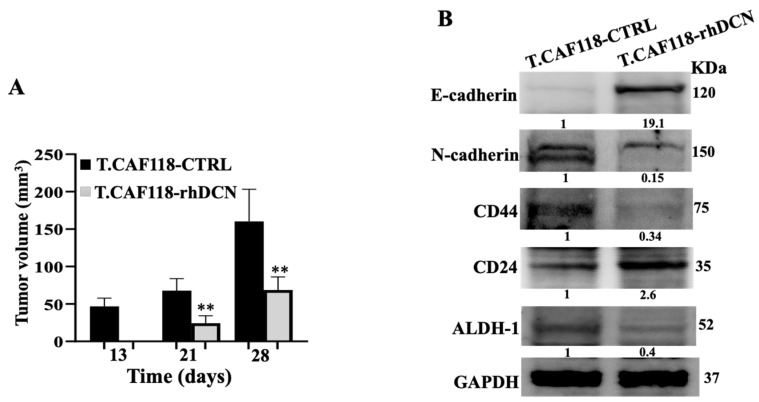
rhDCN-treated CAFs inhibit tumor growth in orthotopic tumor xenografts. Humanized breast cancer orthotopic xenografts were generated by co-injecting MDA-MB-231 cells with either CAF118-CTRL or CAF118-rhDCN cells (T-CAF118-CTRL and T-CAF118-rhDCN, respectively) into the mammary gland of female nude mice (n = 10). (**A**), Tumor volumes, error bars represent mean ± SD (n = 4), ** *p* ≤ 0.01. (**B**), Whole cell lysates from excised tumors were subjected to immunoblotting, and band intensities were normalized to GAPDH and presented as fold changes relative to T-CAF118-CTRL.

To further show the inhibitory effect of rhDCN on CAFs, we have examined the effect of rhDCN on the inhibition of the cancer-promoting effects of CAFs on BC cells in vivo. To test this, humanized orthotopic tumor xenografts were created by injecting MDA-MB-231 cells in combination with either CAF118-CTRL or with CAF118-rhDCN cells (T-CAF118-CTRL and T-CAF118-rhDCN, respectively) under the nipple of nude mice (n = 5 for each inoculation). Both inoculations generated tumors in four out of five animals. Figure 6A shows that only T-CAF118-CTRL formed palpable tumors 13 days after co-injection. Furthermore, tumors bearing CAF118-CTRL cells grew much faster than those containing CAF118-rhDCN cells (Figure 6A). Next, whole cell lysates were prepared from tumors containing CAF118-CTRL and CAF118-rhDCN. Figure 6B shows that rhDCN-treated-CAF-118 cells inhibited the pro-EMT process in breast cancer cells in vivo through increasing the E-cadherin level (19-fold) and decreasing the N-cadherin level (6-fold). In addition, the paracrine pro-stemness process was also inhibited in tumors bearing CAF118-rhDCN cells, via the downregulation of ALDH-1 and CD44 proteins and the upregulation of CD24 (Figure 6B). This indicates that treating CAF cells with rhDCN suppresses their pro-carcinogenic and pro-tumor growth capacities in vivo.

## 4. Discussion

Cytokines, important cell-to-cell messengers, play major roles in inflammation, a process that is a significant pro-carcinogenic factor. On the other hand, there are several anti-carcinogenic cytokines secreted from various types of cells, especially from normal stromal fibroblasts [23]. Thereby, some of these cytokines could be of great therapeutic value as natural anti-cancer molecules [8,24]. Since CAFs are active cancer-promoting cells, we sought in the present report to delineate the effect of DCN, a pan-receptor tyrosine kinase inhibitor with tumor suppressor functions [12], on active breast CAFs. We have shown that rhDCN normalizes the active features of different active breast CAFs. Treatment of CAF cells with rhDCN concurrently upregulated the endogenous DCN and downregulated the CAF biomarkers α-SMA, FAP-α, TGF-β, SDF-1, and IL-6 in cells that were grown in 2D and 3D cultures. These findings prompted us to ask how DCN regulates all these biomarkers. To address this question, we have tested the effect of rhDCN on the expression of AUF-1, which is a positive regulator of all these genes and plays a significant role in the activation of breast stromal fibroblasts [21]. We have found that rhDCN reduced the level of AUF-1 and inhibited its upstream activator STAT3. Thereby, rhDCN suppresses the CAF biomarkers through inhibition of the STAT3/AUF-1 pathway. Regarding TGF-β, it has been shown that rhDCN can bind and sequester the protein [18]. Furthermore, rhDCN downregulates TGF-β in hypertrophic scar fibroblasts [25].

It is well known that DCN inhibits several signaling pathways that are linked to the hallmarks of cancer, which makes DCN a powerful signaling molecule [15,16]. In fact, our LC/MS proteomics analysis has shown that rhDCN can modulate the expression of hundreds of proteins belonging to different pathways related to CAF activation. The inhibitory effect on the NF-κB/STAT3 and ERK1/2 pathways was confirmed by immunoblotting, suggesting that DCN can suppress carcinogenesis by inhibiting these important pro-carcinogenic signaling pathways. Indeed, rhDCN inhibited the proliferative and invasive capacities of active CAFs. This was confirmed at the molecular level by showing that rhDCN suppresses the proliferation and pro-invasive proteins MMP-9 and MMP-2 in active CAF cells [22,26]. It has been previously shown that DCN, as a single agent or in combination with anilinoquinazoline molecule derivatives, inhibits mammary carcinoma cell proliferation and invasion in a dose-dependent manner [27]. Furthermore, Zhang et al. have previously reported that rhDCN inhibits cell proliferation in hypertrophic scar fibroblasts [25]. DCN is associated with the regulation of cancer growth through inhibition of the EGFR and/or TLR2/TLR4 signaling pathways [15]. The interaction of DCN with the EGFR receptor leads to an increase in the expression of p21, which is a tumor suppressor and cell cycle checkpoint protein, resulting in cell cycle arrest [19]. In accordance with these data, we have demonstrated that DCN upregulates both p21 and p16 in DCN-treated CAFs. In addition, Reed et al. have shown that intratumoral injection of rhDCN significantly suppresses primary tumor growth of orthotopic breast carcinoma xenografts [28]. These findings indicate that rhDCN represses the invasive and proliferative abilities of different types of cells. The fact that DCN can inhibit all these pathways and processes shows that the anti-cancer effects of DCN on CAFs are not specific to a particular type of these microenvironmental pro-carcinogenic cells.

The rhDCN-inhibitory effect on active CAFs was further supported by showing suppression of their paracrine pro-carcinogenic effects both in vitro and in orthotopic tumor xenografts. Indeed, we have found that SFCM from rhDCN-treated CAF cells suppressed the pro-EMT process of CAFs. We have also shown that rhDCN-treatment of CAFs inhibited their paracrine promotion of stemness in BC cells, through downregulation of ALDH-1 and CD44 and upregulation of CD24, both in vitro and in tumor xenografts. These effects were further confirmed in a direct co-culture setting. These findings have demonstrated the ability of rhDCN to block the non-cell-autonomous pro-tumorigenic effects of active breast CAFs. Together, these findings provide clear evidence that rhDCN can normalize the active features of CAFs and inhibit their paracrine cancer-promoting effects.

Importantly, rhDCN-dependent inhibition of active CAFs persisted even after the cells were split four times, raising an important question about the molecular basis of this surprising persistence. To address this, we have shown that the *AUF-1* level remained decreased in rhDCN-treated CAF cells. AUF-1 is the positive regulator of all active CAF-biomarkers and is a major component of the IL-6/STAT3/NF-κB positive feedback loop, which maintains CAF permanently active even when these cells are separated from cancer cells [21]. This suggests that rhDCN persistently inhibits active CAFs through AUF-1 downregulation and the consequent blockage of the positive feedback loop. This persistent normalization of active CAFs is not restricted to DCN but has been previously observed in response to natural diet products, namely caffeine and curcumin [29,30]. This further highlights the importance of DCN in treating tumors by normalizing their active CAFs and a “drying up sources” process. In fact, several cytokines have been used for cancer treatment, with IFN-α and IL-2 being the first to be utilized as anti-cancer drugs [7,31]. OPG, which is also secreted from NBFs, was tested for the treatment of BC patients, and it has shown a decrease in serum markers of bone resorption [32]. However, using these cytokines in cancer treatment has serious limitations, such as the use of high doses, which correlated with severe toxicity [33]. Therefore, all these cytokines must be used either modified or in combination with existing therapies to enhance their anti-cancer effectiveness and/or to minimize their side effects [34,35].

## 5. Conclusions

The ability of rhDCN to restore the endogenous level of the protein in active CAFs with a non-toxic concentration could constitute an efficient natural gene therapeutic approach aiming at curing tumors via normalizing their main active feeders (CAFs) or transforming them into antagonists. These findings warrant in vivo confirmation using BC animal models, in order to further prove the potential therapeutic utility of DCN for the treatment of breast tumors.

## Data Availability

The original contributions presented in this study are included in the article/Supplementary Materials. Further inquiries can be directed to the corresponding author.

## Supplementary Materials

The following supporting information can be downloaded at: https://www.mdpi.com/article/10.3390/cells15030311/s1, Figure S1: Proteomics analysis; Figure S2: rhDCN suppresses FAP-α; Figure S3: rhDCN targets breast CSCs biomarkers at the mRNA level; Figure S4: rhDCN inhibitory effects on CAFs are persistent; Table S1: List of primers used for qRT-PCR.

## Abbreviations

The following abbreviations are used in this manuscript:

BC	Breast Cancer
BCSCs	Breast Cancer Stem Cells
CSCs	Cancer Stem Cells
DEPs	Differentially Expressed Proteins
EMT	Epithelial-to-Mesenchymal Transition
CAFs	Cancer-Associated Fibroblasts
SFM	Serum-Free Medium
SFCM	Serum-Free Conditioned Medium
rhDCN	recombinant human DCN
qRT-PCR	quantitative Reverse-Transcription-PCR
