# Supplementary material for: Recombinant Human Decorin Normalizes the Active Features of Breast Cancer-Associated Fibroblasts
Source: Cells. 2026 Feb 6;15(3):311. doi: 10.3390/cells15030311 (PMC12896702; doi:10.3390/cells15030311)
Supplement: Supplementary file 1 [file cells-15-00311-s001.zip › cells-4108823-supplementary.pdf]

SUPPLEMENTARY INFORMATION

Supplementary Table S1. List of primers used for qRT-PCR

Primers	Sequence	
GAPDH	*Forward*	*5'-GAGTCCACTGGCGTCTTC-3'*
	Reverse	*5'-GGGGTGCTAAGCAGTTGGT-3'*
DCN	*Forward*	*5'-TCA AAA GGT CAA GAT CAG CCC-3'*
	Reverse	*5'-CAC TAG CTT TGT GGG CAG TT-3'*
ACTA2 (α-SMA)	*Forward*	*5'-CTATGCCTCTGGACGCACAACT -3'*
	Reverse	*5'-CAGATCCAGACGCATGATGGCA -3'*
CXCL12 (SDF-1)	*Forward*	*5'- CTCAACACTCCAAACTGTGCCC -3'*
	Reverse	*5'-CTCCAGGTACTCCTGAATCCAC-3'*
TGF-β1	*Forward*	*5'-TACCTGAACCCGTGTTGCTCTC -3'*
	Reverse	*5'-GTTGCTGAGGTATCGCCAGGAA -3'*
IL-6	*Forward*	*5'-AGACAG CCA CTC ACC TCT TCA G -3'*
	Reverse	*5'- TTC TGC CAG TGC CTC TTT GCT G -3'*
TWIST1	*Forward*	*5'- GGA CAA GCT GAG CAA GAT TCA GA -3'*
	Reverse	*5'- GTG AGC CAC ATA GCT GCA G -3'*
AUF-1	*Forward*	*5'-GATCAAGGGGTTTTGGCTTT -3'*
	Reverse	*5'-GTTGTCCATGGGGACCTCTA-3'*
FAP-α	*Forward*	*5'-TGA CCA GAA CCA CGG CTT AT -3'*
	Reverse	*5'- AGC AAA CTG TCT GAG GGG TT*
CDH1	*Forward*	*5'-CCC GCC TTA TGA TTC TCT GCT CGT G-3'*
	Reverse	*5'-TCC GTA CAT GTC AGC CAG CTT CTT G-**3'***
CDH2	*Forward*	*5'-CCT CCA GAG TTT ACT GCC ATG AC-3'*
	Reverse	*5'-GTA GGA TCT CCG CCA CTG ATT C-3'*
ALDH1A1	*Forward*	*5'-TCT CTA TTT CTC TCC CCT CCC T-3'*
	Reverse	5'-ACC ATC TTT GAA GGG TTG GC-3'
VEGF-A	*Forward*	5′-CCCACTGAGGAGTCCAACAT-3′
	Reverse	5′-TGGATGGTGGTACAGTCAGAGC-3′
CD24	*Forward*	*5'- GAGAGATAACCCTGCCCGAG-3'*
	Reverse	*5'- AAAAGAAAAGTCCGCGCCTC -3'*
CD44	*Forward*	*5'-CCA GAA GGA ACA GTG GTT TGG C-3'*
	Reverse	*5'-ACT GTC CTC TGG GCT TGG TGT T-3'*

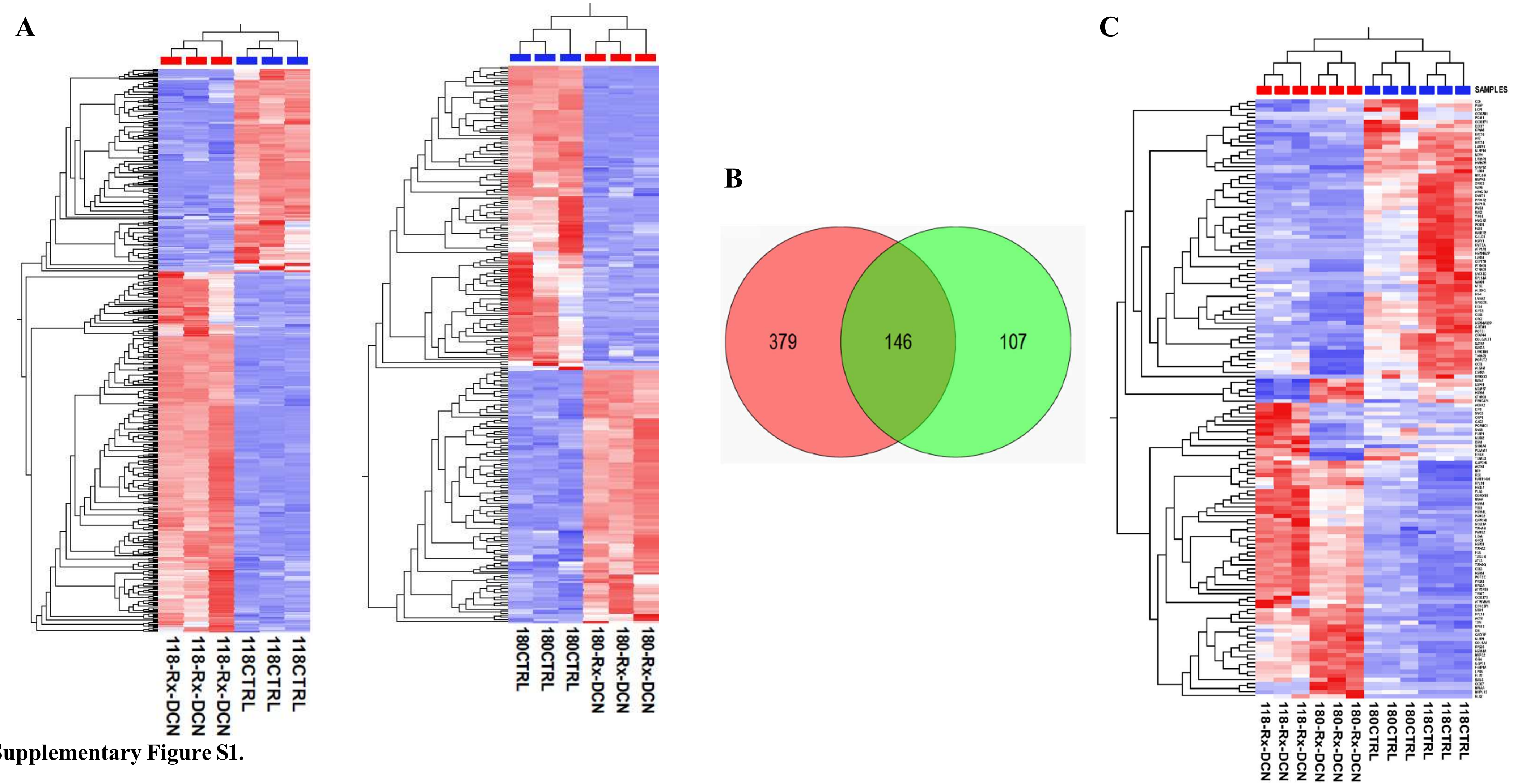

Supplementary Figure S1.

A, Unsupervised Hierarchical cluster analysis of the differentially expressed proteins in the indicated cell cultures. The images were generated using Qlucore Omics Explorer version 3.7, (Lund, Sweden, https://qlucore.com). **B**, Venn diagram analysis and the list of 14 commonly differentially expressed genes. **C**, Unsupervised Hierarchical cluster analysis of the the 146 commonly differentially expressed proteins.

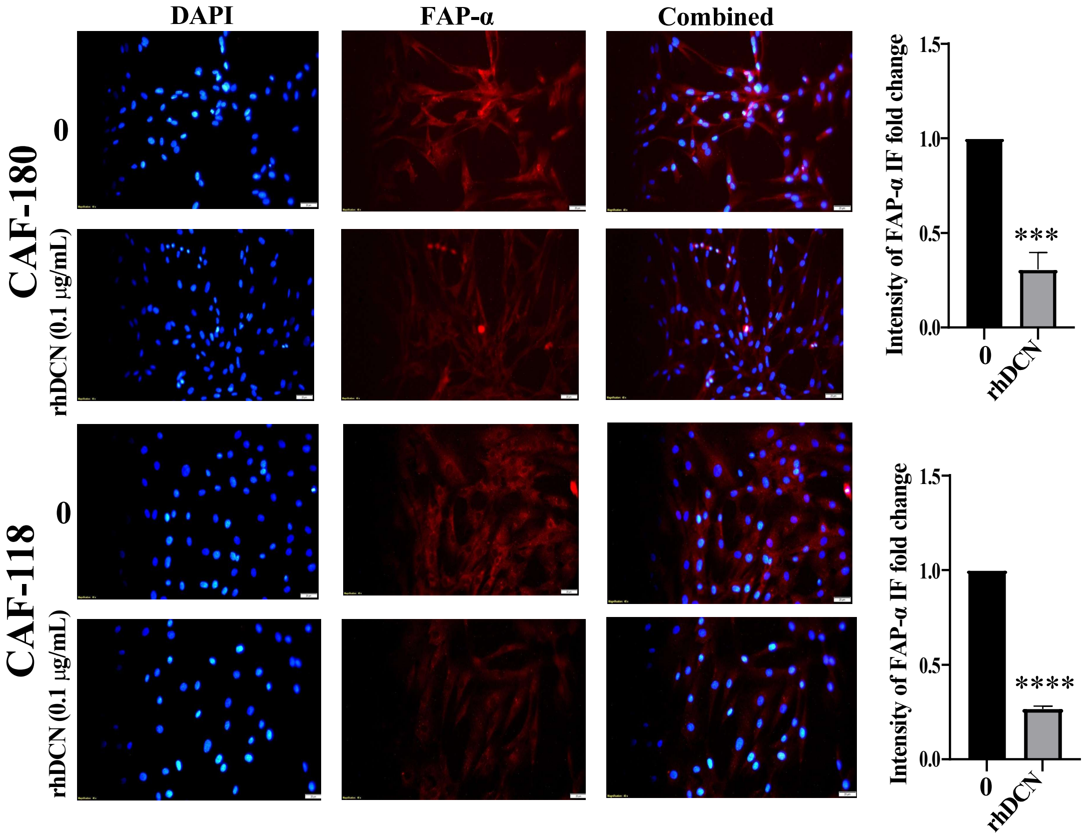

Supplementary Figure S2

CAF cells were treated as shown for 24 h, and then immunofluorescence assay using the indicated antibodies was applied. Left panels: Immunofluorescence microscopy images; right panels: histograms showing fold changes of FAP-α immunofluorescence intensity relative to controls. Error bars represent mean ± SD (n = 3), *** $p \leq 0.001$, **** $p \leq 0.0001$. Scale bars = 20 μm.

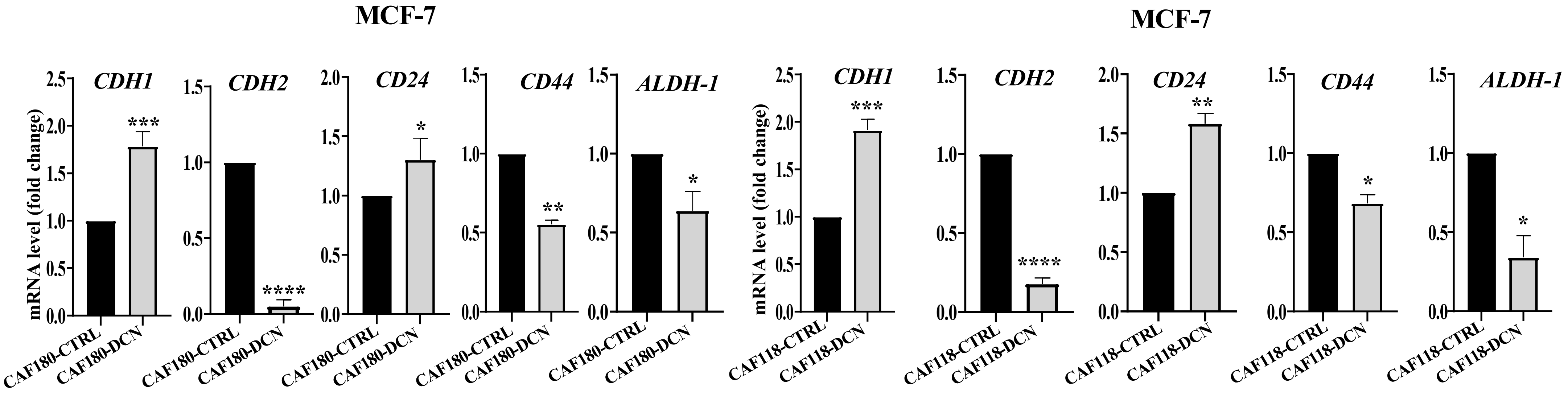

Supplementary Figure S3

CAF-180 and CAF-118 cells were cultured in complete media, and were either sham-treated or challenged with rhDCN (0.1 µg/mL) for 24 h. Then, cells were co-cultured with MCF-7 breast cancer cells using transwell tissue culture plate for 24 h. Total RNA was prepared from MCF-7 cells, and then the mRNAs of the indicated genes were amplified using qRT-PCR. Error bars represent mean ± SD (n = 3). * $p \leq 0.05$, ** $p \leq 0.01$, *** $p \leq 0.001$, **** $p \leq 0.0001$.

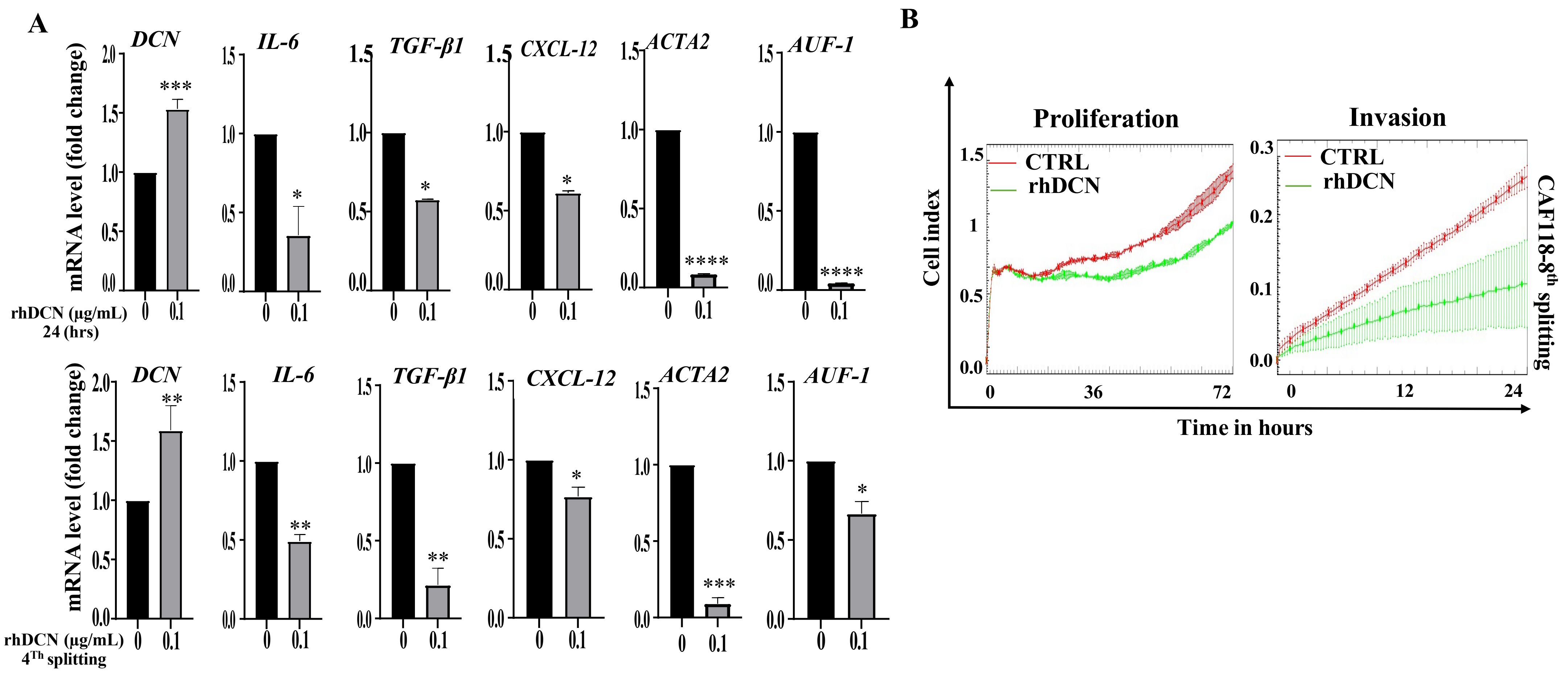

Supplementary Figure S4

A, CAF-118 cells were cultured in complete medium either alone or containing rhDCN (0.1 µg/mL) for 24 h. Each plate was splitted in 2 (one half was used to extract RNA and the other half was re-cultured in complete medium and was splitted three more times). Then, total RNA was prepared, and then the mRNA of the indicated genes was amplified using qRT-PCR. Error bars represent mean ± SD (n = 3). * $p \leq 0.05$, ** $p \leq 0.01$, *** $p \leq 0.001$, **** $p \leq 0.0001$. **B**, Cells invasion and proliferation abilities were assessed using the RTCA-DPxCELLigence System. Data are representative of different experiments performed in triplicate.
